# Research on the application of functional near-infrared spectroscopy in differentiating subjective cognitive decline and mild cognitive impairment

**DOI:** 10.3389/fnagi.2024.1469620

**Published:** 2024-12-24

**Authors:** Zheng Wang, Chaojie Niu, Yong Duan, Hao Yang, Jinpeng Mi, Chao Liu, Guodong Chen, Qihao Guo

**Affiliations:** ^1^Department of Gerontology, Shanghai Sixth People’s Hospital Affiliated to Shanghai Jiao Tong University School of Medicine, Shanghai, China; ^2^Jiangsu Provincial Key Laboratory of Advanced Robotics, and Robotics and Microsystems Center, School of Mechanical and Electrical Engineering, Soochow University, Suzhou, China; ^3^School of Mechanical and Automotive Engineering, Shanghai University of Engineering Science, Shanghai, China; ^4^Institute of Machine Intelligence (IMI), University of Shanghai for Science and Technology, Shanghai, China

**Keywords:** functional near-infrared spectroscopy, MCI, SCD, Alzheimer’s disease, early diagnosis

## Abstract

**Introduction:**

Alzheimer’s disease (AD) is a common neurological disorder. Based on clinical characteristics, it can be categorized into normal cognition (NC), subjective cognitive decline (SCD), mild cognitive impairment (MCI), and dementia (AD). Once the condition begins to progress, the process is usually irreversible. Therefore, early identification and intervention are crucial for patients. This study aims to explore the sensitivity of fNIRS in distinguishing between SCD and MCI.

**Methods:**

An in-depth analysis of the Functional Connectivity (FC) and oxygenated hemoglobin (HbO) characteristics during resting state and different memory cognitive tasks is conducted on two patient groups to search for potential biomarkers. The 33 participants were divided into two groups: SCD and MCI.

**Results:**

Functional connectivity strength during the resting state and hemodynamic changes during the execution of Verbal Fluency Tasks (VFT) and MemTrax tasks were measured using fNIRS. The results showed that compared to individuals with MCI, patients with SCD exhibited higher average FC levels between different channels in the frontal lobe during resting state, with two channels’ FC demonstrating significant ability to distinguish between SCD and MCI. During the VFT task, the overall average HbO concentration in the frontal lobe of SCD patients was higher than that of MCI patients from 5 experimental paradigm. Receiver operating characteristic analysis indicated that the accuracy of the above features in distinguishing SCD from MCI was 78.8%, 72.7%, 75.8%, and 66.7%, respectively.

**Discussion:**

fNIRS could potentially serve as a non-invasive biomarker for the early detection of dementia.

## 1 Introduction

Alzheimer’s disease (AD) is currently the seventh leading cause of death globally and one of the primary reasons for the loss of independence and reliance on others among the elderly (World Health Organization, 2023). In 2019, the global societal total cost of AD was 1.3 trillion USD; by 2030, with the rising prevalence, the cost is expected to reach 2.8 trillion USD. Depending on the degree of cognitive impairment, AD can be divided into several stages: normal cognition (NC), subjective cognitive decline (SCD), mild cognitive impairment (MCI), and AD (Jak et al., 2009). Although SCD and MCI do not inevitably progress to AD, they are both high-risk groups for AD, especially those patients who have already exhibited AD pathological characteristics, considered as the preclinical stage of AD (Ehlis et al., 2014; Petersen, 2016). Given that early treatment is more effective, developing a simple, reliable, and sensitive biomarker for early diagnosis in high-risk populations has significant clinical importance.

Currently, there are various diagnostic methods for the AD spectrum. Clinical diagnosis mainly relies on patient history and neuropsychological tests, such as the Mini-Mental State Examination (MMSE) (Folstein et al., 1975) and Montreal Cognitive Assessment (Nasreddine et al., 2005). However, neuropsychological tests are susceptible to factors such as education and age and require experienced physicians to administer (Nguyen et al., 2008). To address this issue, the National Institute on Aging and the Alzheimer’s Association have proposed the latest diagnostic guidelines,^1^ suggesting that the diagnostic criteria for AD should be defined from a biological perspective rather than clinical symptoms. Previous research has identified a series of biomarkers, including the use of positron emission tomography (PET) to detect the accumulation of beta-amyloid (Aβ) and tau proteins, as well as reduced glucose metabolism in the brain (Jack et al., 2018; Xie et al., 2022). Additionally, magnetic resonance imaging (MRI) can be used to assess changes in hippocampal volume, while diffusion MRI is used to observe the structural integrity of brain white matter (Gerischer et al., 2018; Ten Kate et al., 2024). However, the aforementioned biomarkers are either invasive (cerebrospinal fluid and PET) (Dubbelman et al., 2024) or involve large equipment and complex procedures (PET and MRI). They are costly and have low accessibility, making them difficult to widely apply.

Functional near-infrared spectroscopy (fNIRS) is a wearable, portable, non-invasive, low-cost, and high temporal resolution (compared to fMRI) optical neuroimaging technique (Ferrari and Quaresima, 2012; Quaresima et al., 2012; Torricelli et al., 2014). It uses two or more near-infrared lights to estimate changes in neuronal activity caused by variations in the concentration of oxygenated hemoglobin (HbO) and deoxygenated hemoglobin (HbR) (Bondi et al., 2014; Ehlis et al., 2014). Multiple studies have shown that compared to healthy control groups, SCD, MCI, and AD patients exhibit a more severe reduction in frontal lobe activation during certain cognitive tasks, with MCI showing a more pronounced reduction compared to SCD, and the severity of AD pathology being related to the extent of HbO abnormalities (Yoon et al., 2019; Yeung and Chan, 2020; Zhang et al., 2024). Many studies have also explored the possibility of using fNIRS to detect different biomarkers for diagnosing AD, where appropriate biomarkers can provide reliable diagnostic results for patients (Li R. et al., 2018; Li X. et al., 2018; Zhang et al., 2023).

In the early diagnosis and intervention research of AD, although current research focuses mainly on the comparative analysis between NC and MCI to discover potential biomarkers indicating the development of AD (Yap et al., 2017; Wang et al., 2024), studies on the transitional stage between SCD and MCI are relatively scarce. Individuals with SCD exhibit a subjective perception of decline in cognitive function relative to their previous normal state, even though this decline does not yet meet the clinical diagnostic criteria for MCI (Esmaeili et al., 2022; Li et al., 2022). Previous studies have found that Reisberg and Gauthier (2008) speculated that SCD precedes MCI, belonging to the symptomatic pre-dementia stage of AD, and he estimated that up to 60% of SCD patients will develop into MCI and AD within 15 years. Furthermore, Jessen (2014), Molinuevo et al. (2017), and others have proposed that the early risk signs of AD may be the feeling of decline in one or more cognitive domains experienced by patients with SCD compared to their previous state, suggesting a certain correlation between SCD and AD pathological changes. Although the neuropsychological test results of SCD patients are still within the normal range, their brain structure may have already undergone some minor degenerative changes (Amariglio et al., 2012; Wang et al., 2020; Jia et al., 2021; Schwarz et al., 2021). Increasing evidence indicates (Jessen, 2014) that the likelihood of elderly individuals with SCD exhibiting biomarker abnormalities consistent with AD pathology increases, as does the future risk of pathological cognitive decline and dementia.

Therefore, the SCD stage provides a critical observation window for understanding the early pathological changes of AD, and its importance in early diagnosis and intervention strategies should not be overlooked (Pike et al., 2022; Ulbl and Rakusa, 2023). Future research should strengthen the exploration of the transitional stage between SCD and MCI to discover new biomarkers, providing a more solid scientific foundation for the early diagnosis and intervention of AD. SCD, as the precursor stage to MCI, offers an important opportunity for early diagnosis and intervention, which is crucial for timely monitoring and early treatment, especially in the preclinical stages of neurocognitive disorders such as AD (Lin et al., 2019; Esmaeili et al., 2022). By identifying SCD, effective stratification of high-risk populations can be implemented, allowing for the deployment of preventive strategies and targeted intervention measures aimed at intervening early in the disease’s progression to slow the transition to MCI and subsequent dementia syndromes. Finding relevant biomarkers is essential for the prevention and treatment of the progression from SCD to MCI and AD.

The objective of this study is to utilize fNIRS technology as a tool to further advance the early detection of individuals with SCD. For participants with different degrees of illness, we propose a comprehensive experimental scheme, including resting, cognitive, memory, and language tests for in-depth analysis, to search for potential biomarkers. Subsequently, stepwise binary logistic regression analysis will be used to assess whether those biomarkers with significant intergroup differences have the potential to differentiate between different stages of AD.

## 2 Materials and methods

### 2.1 Participants

**FIGURE 1:**
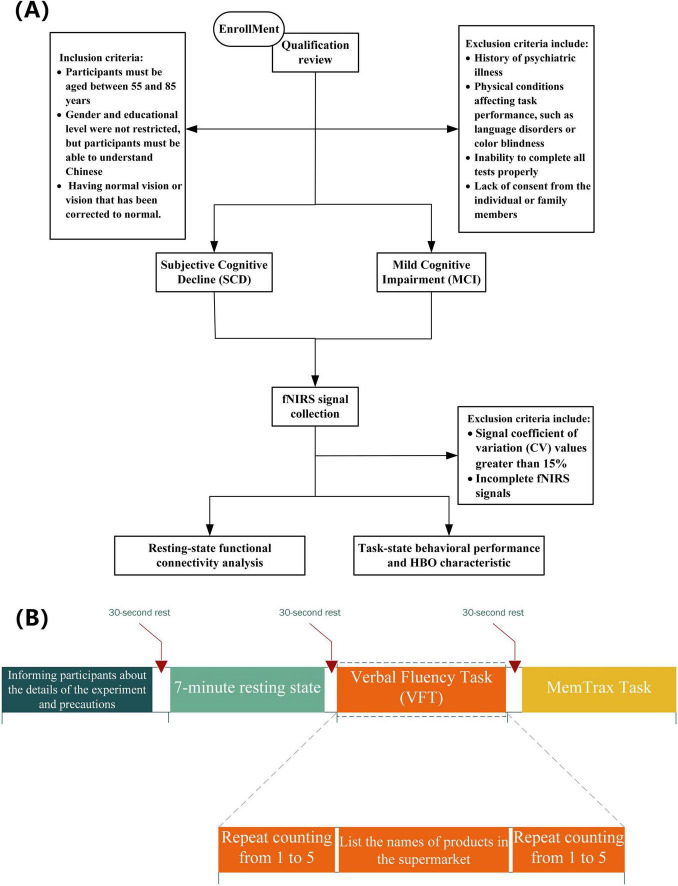
**(A)** Overall research process flowchart. **(B)** Experimental paradigm.

This study recruited a total of 33 adults. The inclusion criteria were as follows: (1) participants must be aged between 55 and 85 years; (2) gender and educational level were not restricted, but participants must be able to understand Chinese and possess normal or corrected-to-normal vision; (3) individuals with a history of psychiatric disorders or other physical illnesses that could affect task performance (such as language disorders or color blindness) were excluded; (4) volunteers unable to complete the experimental tasks were also excluded. Figure 1 illustrates the entire research screening and analysis process. All volunteers were recruited from the Shanghai Sixth People’s Hospital. This experiment was approved by the Clinical Trial Ethics Committee of Shanghai Sixth People’s Hospital, and all participants were informed and consented to participate in the study prior to their involvement. Grouping criteria: For the SCD group, according to the diagnostic criteria established by Jessen et al. (2020), participants met five conditions: subjective memory decline; onset time of less than 5 years; concern about cognitive decline; onset after the age of 55; and no significant objective cognitive impairment compared to individuals with normal cognition (NC) in neuropsychological tests, with cognitive deficits not meeting the criteria for MCI (Rabin et al., 2017). For the MCI group, cognitive impairment met the Jak/Bondi criteria (Bondi et al., 2014).

### 2.2 Procedures

All participants underwent a standardized neuropsychological assessment. The assessment included tests for memory, executive function, attention, language, and overall cognitive abilities. The assessment tools comprised the MMSE, Shape Trail Test (Zhao et al., 2013), and Addenbrooke’s Cognitive Examination III (ACE-III) (Wang et al., 2017). Additionally, the Boston Naming Test (Williams et al., 1989), category fluency test (Chan and Poon, 1999), and Montreal Cognitive Assessment-Basic (MOCA-B)(Nasreddine et al., 2005) were administered. Subsequently, functional near-infrared spectroscopy (fNIRS) data collection was conducted. During the fNIRS data acquisition, participants were required to perform pre-established tasks while their frontal lobe hemodynamic activities were recorded. Participants were instructed to remain still and minimize head and body movements to prevent any unnecessary motion artifacts. These comprehensive assessments and data collection steps were designed to thoroughly evaluate the cognitive status of the participants and provide a solid foundation for subsequent analysis.

### 2.3 Experimental paradigm

Participants must be seated in a relaxed posture within an enclosed chamber to attenuate extraneous environmental interference, and they are advised to eschew any unnecessary corporeal motion. In this study, each group of subjects will sequentially participate in three parts of the test, including a resting state task, a Verbal Fluency Task (VFT), and the MemTrax task (Liu et al., 2024). Subjects have 30 s of rest before the start of each part of the task. During the resting state task, subjects need to sit quietly with their eyes closed, try to maintain a relaxed state, and avoid thinking and falling asleep. The recording of the resting state will last for 7 min without any prior warning. After 7 min, the recording signal is checked for completeness, and the subjects are informed that the test has been completed. This is followed by the VFT, which includes three stages: (1) participants continuously repeat counting from 1 to 5 within 30 s to obtain a baseline value for cognitive performance; (2) participants are asked to recall and say the names of items in a supermarket as much as possible, lasting 60 s; (3) participants continuously repeat step 1 within 30 s to return to the baseline level. Finally, the MemTrax task is conducted, where participants will see a series of pictures on the computer screen. When they see the same picture appearing repeatedly, they must quickly press the spacebar. The system will record the participants’ reaction time and accuracy rate. Participants who do not complete these tasks in full will be excluded from the final analysis.

### 2.4 Hemodynamic response

To measure the hemodynamic activity in the prefrontal cortex under different task conditions, we used a 19-channel continuous fNIRS NirSmart system (Danyang Huichuang Medical Equipment Co., Ltd., China) to collect fNIRS data from the prefrontal lobe. The system employs near-infrared light with wavelengths of 730 and 850 nm, using the modified Beer–Lambert law to estimate the relative concentration of HbO in the participants’ prefrontal cortex. The fNIRS system consists of 7 light sources and 7 detectors arranged in a 2 × 7 matrix configuration, with a 3 cm distance between each source and detector. The center of the bottom probe is approximately located at FpZ, following the international 10/20 system. The sampling rate of the NirSmart system is set to 11 Hz.

### 2.5 Data pre-processing

Before formally analyzing the data, it is necessary to first verify the integrity and reliability of the data. Initially, we check whether the fNIRS data of the 33 participants is complete, ensuring that the fNIRS data for each task has sufficient experimental duration and valid starting labels. Since the fNIRS signal may be affected by changes in the signal-to-noise ratio (SNR), we use the coefficient of variation (CV) to exclude channels with poor signal quality. The formula for calculating the CV value is as follows:


(1) C⁢V=σ/μ

where σ and μ are the standard deviation and mean value of the fNIRS data in the same channel, respectively. Channels with a CV greater than 15% will be rejected for further use, as a higher CV value usually indicates a lower SNR (Piper et al., 2014).

The fNIRS data is preprocessed using NirSpark software (Danyang Huichuang Medical Equipment Co., Ltd., China). Motion artifacts caused by relative sliding of the scalp and probe are corrected using moving standard deviation and spline interpolation methods (SDThresh = 20, AMPThresh = 3, tMotion = 0.5 s, tMask = 1 s, and *p* = 0.99). Subsequently, a bandpass filter with a cutoff frequency of 0.01–0.1 Hz is used to eliminate systemic physiological noise (including respiration, cardiac activity, and low-frequency signal drift). The modified Beer–Lambert law is used to convert light intensity into relative concentration changes of hemoglobin. Data recorded 10 s before the start of the task is used as a baseline for baseline correction. Considering that the oxyhemoglobin (HbO) signal has a better SNR than the deoxyhemoglobin (HbR) signal in fNIRS measurements, we mainly focus on the HbO signal for data analysis of all participants’ brain signals.

### 2.6 Data analysis

For the HbO data, considering the 3–5 s delay in hemodynamic response, it is necessary to include the 5 s following task completion in the analysis when using the block averaging method to calculate the mean HbO concentration changes over different time periods. Additionally, we have taken into account the changes in the HbO slope and standard deviation during various intervals of the task. For the VFT, we calculated the average HbO concentration, slope, and standard deviation within the 5–15, 20–60, and 60–70 s time windows, respectively. Regarding the MemTrax task, to facilitate analysis, we aligned the HbO data of all participants with that of the participant with the shortest task duration and similarly calculated the average HbO concentration, slope, and standard deviation within the 5–15 s, 20–60 s, and 60-s to task completion time windows. In the resting state, the FC strength of each pair of measurement channel time series was calculated using Pearson correlation analysis. A two-sample *t*-test and Chi-square test were employed to examine differences between groups in demographic variables, neuropsychological assessment performance, behavioral performance on the MemTrax task, HbO data characteristics under different task conditions, and FC strength in the resting state. Furthermore, stepwise discriminant analysis was performed to assess the factors influencing the differentiation of HbO data between groups. This was followed by a refined analysis using stepwise binary logistic regression to assess the specific impact of each factor on the binary outcome. Subsequently, receiver operating characteristic (ROC) analysis was performed. All statistical analyses were conducted using SPSS 26.0 (IBM Corporation, Armonk, NY, USA). Unless otherwise specified, all tests were set at a significance level of 0.05 (two-tailed).

## 3 Results

### 3.1 Demographic information and neuropsychological assessment performance

Table 1 presents the demographic and neuropsychological assessment performance of the SCD and MCI groups. There were no significant differences between the SCD and MCI groups in terms of gender, age, and education level (*p* = 0.462–0.858). However, significant intergroup differences were observed in various cognitive assessments. Specifically, in the category fluency test (animal naming and alternate city-surname listing), the SCD group had average scores of 17.69 (SD = 3.9) and 23.86 (SD = 7.16), while the MCI group had average scores of 13.05 (SD = 3.56) and 18.45 (SD = 7.42). These results indicate that the SCD group performed significantly better than the MCI group in both category fluency tests (*t*(31) = 3.525, *p* = 0.001; *t*(31) = 2.068, *p* = 0.047). In the ACE-III test, the SCD group had an average score of 78.08 (SD = 7.44), whereas the MCI group had an average score of 69.8 (SD = 9.64). The SCD group’s performance was also significantly better than that of the MCI group (*t*(31) = 2.623, *p* = 0.013). Additionally, the SCD group outperformed the MCI group in other neuropsychological tests, including the Boston Naming Test, MMSE, and Montreal Cognitive Assessment. Furthermore, in the Trail Making Test Part 1, the SCD group spent less time than the MCI group, although these differences were not statistically significant (*p* = 0.828–0.071).

**TABLE 1:** Demographic information and neuropsychological assessment performance.

Characteristics	SCD(*N* = 13)		MCI(*N* = 20)			
**Variables**	**Mean**	**SD**	**Mean**	**SD**	***p*-Value**	**Significance**
**Demographics**						
Gender (M/F)	7/6		9/11		0.619	
Age (years)	69	7.27	69.4	5.46	0.858	
Education (years)	12.31	2.53	11.3	4.41	0.462	
**Boston Naming Test**						
Spontaneous naming	23.08	3.4	21.4	4.62	0.27	
**Category fluency test**						
Animals	17.69	3.9	13.05	3.56	0.001	MCI < SCD
Cities and surnames	23.86	7.16	18.45	7.42	0.047	MCI < SCD
**Shape Trail Test**						
Trails 1 completion time (s)	52.31	12.38	66.15	31.07	0.138	
Trails 2 completion time (s)	182.23	46.24	157.65	53.47	0.184	
MMSE	27.23	1.59	26.2	2.24	0.16	
MOCA-B	24.31	2.93	22.1	3.54	0.071	
ACE-III	78.08	7.44	69.8	9.64	0.013	MCI < SCD

Except for gender, all characteristics are represented by the mean ± standard deviation. Gender is denoted using the male/female format. The *p* values are obtained using the Chi-square test for gender. All other *p* values are derived from independent samples *t*-tests.

### 3.2 Analysis of fNIRS data during resting state

#### 3.2.1 Analysis of functional connectivity during resting state

**FIGURE 2:**
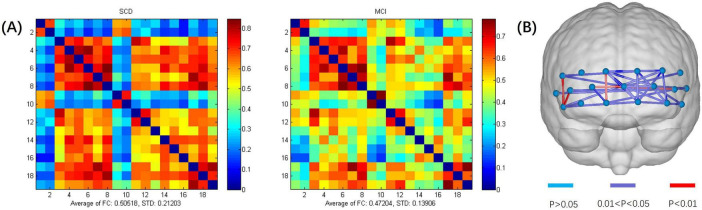
**(A)** Functional connectivity heatmap for the SCD and MCI groups. **(B)** Schematic diagram of channels with significant functional connectivity differences between the two groups. The scale in the image represents the range of values corresponding to the colors. The color gradient transitions from blue to red, indicating a range from low to high values. The numbers at the top and bottom of the scale represent the maximum and minimum values.

An exhaustive analysis of fNIRS data in a resting state revealed no statistically significant differences between the two study groups in terms of average HbO concentration changes, HbO slope, and standard deviation across different time windows. These results suggest that the trends and variability of these physiological parameters are similar across groups in a resting state. However, when examining the FC between different channels in a resting state, significant intergroup differences were found between the SCD and MCI groups, as shown in Figure 2. The scale in the image represents the range of values corresponding to the colors. The color gradient transitions from blue to red, indicating a range from low to high values. The numbers at the top and bottom of the scale represent the maximum and minimum values, respectively. The results indicated that the average FC strength values for the SCD and MCI groups were 0.505 (SD = 0.212) and 0.472 (SD = 0.139), respectively. Compared to the MCI group, the SCD group exhibited a stronger pattern of FC. Combined with the neuropsychological assessment results where the MCI group scored generally lower than the SCD group, this suggests that the weaker connectivity pattern may reflect a trend of brain network degeneration in the progression of neurodegenerative diseases (Lopez-Sanz et al., 2017; Hojjati et al., 2018). This is consistent with previous findings of reduced frontal lobe resting-state FC in MCI patients, indicating that changes in the FC pattern may be a potential mechanism for determining the degree of illness.

After conducting a two-sample *t*-test to statistically analyze the inter-channel connectivity strength between the SCD and MCI groups, significant intergroup differences were found in the FC strength of 29 channel pairs (*p* < 0.05) (Table 2). Of these, 20 pairs showed significantly higher FC strength in the SCD group compared to the MCI group (*p* < 0.05, *t* = 2.09–3.16), while the remaining 9 pairs had significantly lower connectivity strength in the SCD group (*p* < 0.05, *t* = 2.99 to 2.04). Additionally, five pairs of channels exhibited particularly significant differences in FC strength (*p* < 0.01), namely CH1–CH11 (*p* = 0.006, *t* = -2.95), CH1–CH12 (*p* = 0.005, *t* = 2.99), CH4–CH15 (*p* = 0.003, *t* = 3.12), CH5–CH14 (*p* = 0.004, *t* = 3.15), and CH14–CH17 (*p* = 0.009, *t* = 2.79). These findings suggest that changes in FC patterns could potentially serve as biomarkers for determining the degree of illness. In-depth study of resting-state brain FC could contribute to the neurobiological basis of SCD and MCI and provide important evidence for early diagnosis of the disease.

**TABLE 2:** Different group mean HbO channel-to-channel connectivity strength.

Channel-to-channel	Connectivity strength				*t*-Value	*p*-Value
	**SCD mean**	**SCD SD**	**MCI mean**	**MCI SD**		
1–3	0.33	0.27	0.52	0.26	2.06	0.047
1–6	0.19	0.30	0.40	0.26	2.17	0.038
1–11	0.29	0.25	0.54	0.24	2.95	0.006*
1–12	0.24	0.25	0.51	0.25	2.99	0.005*
1–18	0.17	0.22	0.34	0.25	2.04	0.050
2–10	0.67	0.22	0.46	0.25	2.45	0.020
4–5	0.80	0.10	0.64	0.32	2.10	0.047
4–15	0.68	0.17	0.40	0.29	3.12	0.004*
4–17	0.66	0.14	0.50	0.29	2.19	0.037
5–14	0.69	0.14	0.38	0.40	3.16	0.004*
6–9	0.25	0.23	0.47	0.28	2.33	0.027
6–14	0.75	0.24	0.53	0.34	2.11	0.043
6–15	0.64	0.19	0.43	0.31	2.22	0.034
6–16	0.71	0.17	0.53	0.31	2.16	0.039
6–18	0.77	0.12	0.62	0.27	2.21	0.035
7–14	0.58	0.18	0.39	0.34	2.09	0.045
7–16	0.58	0.20	0.36	0.32	2.21	0.035
7–18	0.73	0.13	0.54	0.29	2.19	0.036
8–9	0.27	0.23	0.46	0.23	2.37	0.024
8–10	0.17	0.22	0.37	0.26	2.21	0.034
8–14	0.73	0.17	0.49	0.36	2.58	0.015
8–15	0.67	0.18	0.47	0.30	2.44	0.021
10–11	0.21	0.27	0.43	0.31	2.10	0.044
12–14	0.55	0.20	0.34	0.35	2.23	0.033
12–16	0.52	0.21	0.28	0.40	2.24	0.033
14–15	0.69	0.18	0.42	0.42	2.46	0.020
14–17	0.67	0.18	0.43	0.31	2.79	0.009*
14–19	0.63	0.24	0.38	0.33	2.41	0.022
15–18	0.66	0.19	0.44	0.30	2.33	0.026

SCD, subjective cognitive decline; MCI, mild cognitive impairment.

*p < 0.01.

#### 3.2.2 Discriminant analysis of SCD and MCI during resting state

To evaluate the ability of fNIRS to distinguish between SCD and MCI in a resting state, discriminant analysis was performed using the connectivity strength of 29 channel pairs as features. To identify the optimal features and reduce the dimensionality of classification, a preliminary selection was conducted among the channel pairs with significant intergroup differences. The connectivity strength values of the five channel pairs with the most significant differences were used as input features for stepwise discriminant analysis. This method aids in identifying the most influential features for classification, thus enhancing the model’s predictive accuracy. It was found that CH1–CH12 and CH4–CH15 had more significant classification effects in differentiating between SCD and MCI. Subsequent stepwise binary logistic regression tests of these two channel pairs confirmed that CH1–CH12 and CH4–CH15 are significant predictors in the SCD and MCI classification model, explaining 21.9% and 24.9% of the variance in group membership, respectively (Cox and Snell *R*^2^ = 0.219, 0.249). The classification accuracy rates were 78.8% and 72.7%, respectively. Additionally, the goodness of fit for CH1–CH12 (χ^2^(8) = 6.448, *p* = 0.597) and CH4–CH15 (χ^2^(8) = 4.633, *p* = 0.796) was confirmed through the Hosmer and Lemeshow test. The *p* values for the Hosmer and Lemeshow test for both significant predictors were greater than 0.05, indicating that the model has a good fit and is suitable for this classification task.

**FIGURE 3:**
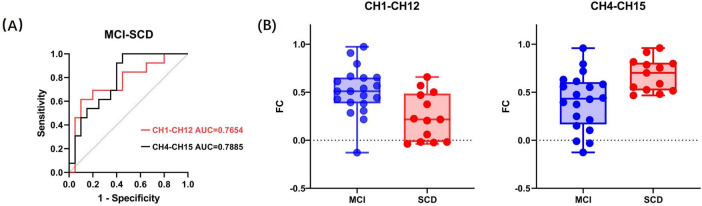
**(A)** Receiver operating characteristic curves of the two significant factors. **(B)** The distribution of the two significant factors within the MCI and SCD groups. FC refers to functional connectivity.

Further ROC analysis showed that the area under the ROC curve (AUC) for CH1–CH12 was 0.7654 (*p* = 0.011), and for CH4–CH15, it was 0.7885 (*p* = 0.006). The distribution of these two significant factors across different groups is displayed in Figure 3. FC refers to functional connectivity. The ROC analysis also revealed cutoff values for the FC strength of CH1–CH12 (0.515, sensitivity = 0.9, specificity = 0.615) and CH4–CH15 (0.45, sensitivity = 1, specificity = 0.55). Specifically, since the *t*-value of the two-sample *t*-test for CH1–CH12 FC strength between the SCD and MCI groups was negative, and for CH4–CH15, it was positive (with SCD as the first group and MCI as the second group), it can be inferred that the average FC strength in CH1–CH12 was lower in the SCD group than in the MCI group, while in CH4–CH15, it was higher in the SCD group. Consequently, when the FC strength between CH1 and CH12 channels falls below the cutoff value, participants are classified as SCD. Conversely, when the FC strength between CH4 and CH15 channels exceeds the cutoff value, participants are classified as SCD.

It is noteworthy that even after including demographic factors such as age, gender, and education level in the statistical model, only CH1–CH12 and CH4–CH15 were selected as significant predictors. This result suggests that the FC strength between CH1–CH12 and CH4–CH15 is independently associated with the differentiation between SCD and MCI, beyond the influence of demographic factors

### 3.3 Analysis of fNIRS data during Verbal Fluency Task

#### 3.3.1 Hemodynamic data analysis during VFT

In this study, we specifically examined the average HbO concentration and its derivative features (slope and standard deviation) within various time windows (5–15, 20–60, 60–70, and 5–65 s) in the SCD and MCI groups to identify physiological differences between these cognitive states. Through meticulous comparative analysis, we aimed to uncover potential mechanisms of cerebral hemodynamics during the VFT in patients with SCD and MCI.

The division of time windows was primarily intended to detect significant intergroup differences by examining the trends and latency characteristics of hemodynamic responses during different periods. Considering the 3–5 s latency in hemodynamic responses, the time windows were categorized as follows: (1) initial increase interval of average HbO concentration (5–15 s): this phase reflects the preliminary rise in HbO concentration following the commencement of the task; (2) plateau phase of average HbO concentration during the task (20–60 s): in this interval, HbO concentration tends to stabilize, indicating the influence of sustained cognitive activity; (3) final decrease phase of average HbO concentration post-task (60–70 s): after the task concludes, HbO concentration gradually reverts to baseline levels; (4) average HbO concentration throughout the task duration (5–65 s): this comprehensive time window provides an overview of the overall HbO concentration changes.

**FIGURE 4:**
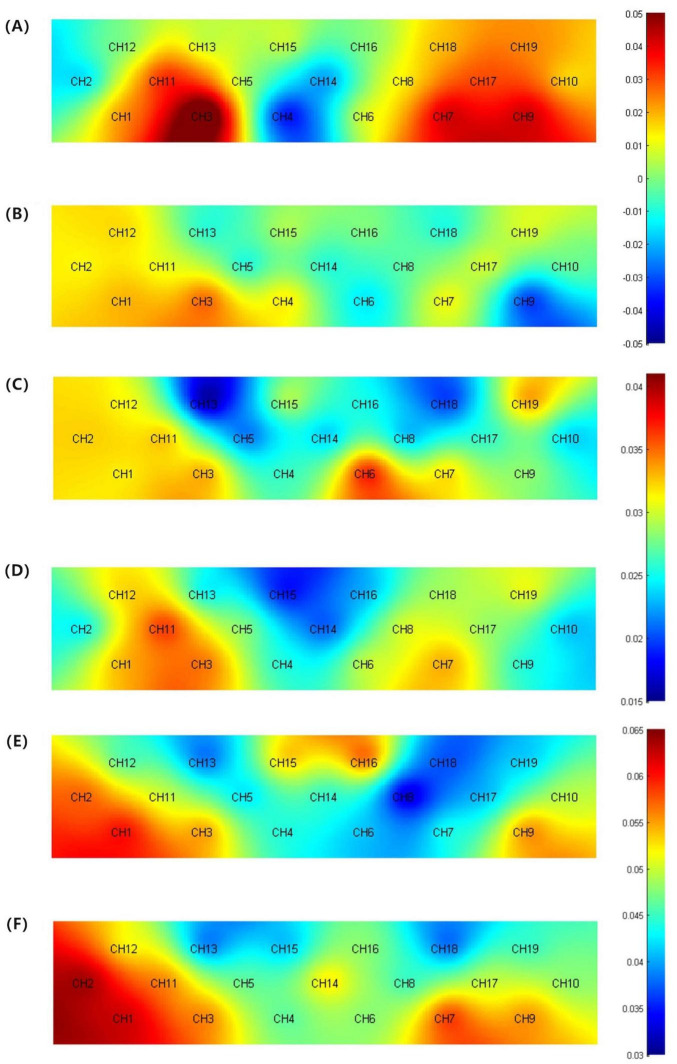
**(A)** Activation map of average HbO concentration for the SCD group (5–15 s). **(B)** Activation map of average HbO concentration for the MCI group (5–15 s). **(C)** Topographic map of standard deviation for the SCD group (5–15 s). **(D)** Topographic map of standard deviation for the MCI group (5–15 s). **(E)** Topographic map of standard deviation for the SCD group (20–60 s). **(F)** Topographic map of standard deviation for the SCD group (20–60 s).

Analysis of the average HbO concentration and its derivative features across the four time windows revealed that during the VFT, there were no significant differences between the two groups in the 20–60, 60–70, and 5–65 s intervals. This suggests a certain similarity in cerebral hemodynamic responses between the groups during these stages. However, within the 5–15 s window, channels CH3 and CH7 exhibited slightly higher average HbO concentrations compared to the MCI group, though not significantly different (*p* > 0.05). In contrast, channel CH9 showed a significantly higher average HbO concentration compared to the MCI group (*t*(31) = 3.29, *p* = 0.02). This finding may indicate that, compared to MCI patients, those with SCD exhibit stronger cerebral activity at the onset of the task. Regarding standard deviation, there were no significant intergroup differences in the 5–65 and 60–70 s windows (*p* > 0.05). However, significant intergroup differences were observed in the standard deviation of channel CH13 within the 5–15 s window and channel CH8 within the 20–60 s window. Specifically, the SCD group’s standard deviation was significantly lower than that of the MCI group (*t*(31) = 2.353, *p* = 0.025; *t*(31) = 2.381, *p* = 0.024), detailed in Figure 4. These results suggest that in specific channels and timeframes, the cerebral hemodynamic responses of SCD patients demonstrate greater consistency and concentration. This may indicate that during cognitive tasks, SCD patients have relatively smaller fluctuations in HbO levels, showing higher stability, whereas MCI patients exhibit larger fluctuations and less stability under the same conditions.

#### 3.3.2 Discriminant analysis of SCD and MCI in VFT

In this study, we specifically investigated the ability of fNIRS during the VFT to differentiate between SCD and MCI by considering features that exhibited significant intergroup differences. We evaluated the following features within specific time windows: average HbO concentration in the 5–15 s window, HbO standard deviation in the 5–15 s window, and HbO concentration standard deviation in the 20–60 s window. Due to the limited number of statistical features, a secondary selection was not necessary.

The results indicated that the HbO standard deviation of channel CH13 within the 5–15 s window had only a marginal impact on classification (*p* = 0.055). However, the average HbO concentration of channel CH9 in the 5–15 s window and the HbO concentration standard deviation of channel CH8 in the 20–60 s window emerged as significant predictors in the SCD-MCI classification model (*p* < 0.05). These two significant predictors explained 29.6% and 21.6% of the variance in group membership, respectively (Cox and Snell *R*^2^ = 0.296, 0.216), with classification accuracies of 75.8% and 66.7%.

**FIGURE 5:**
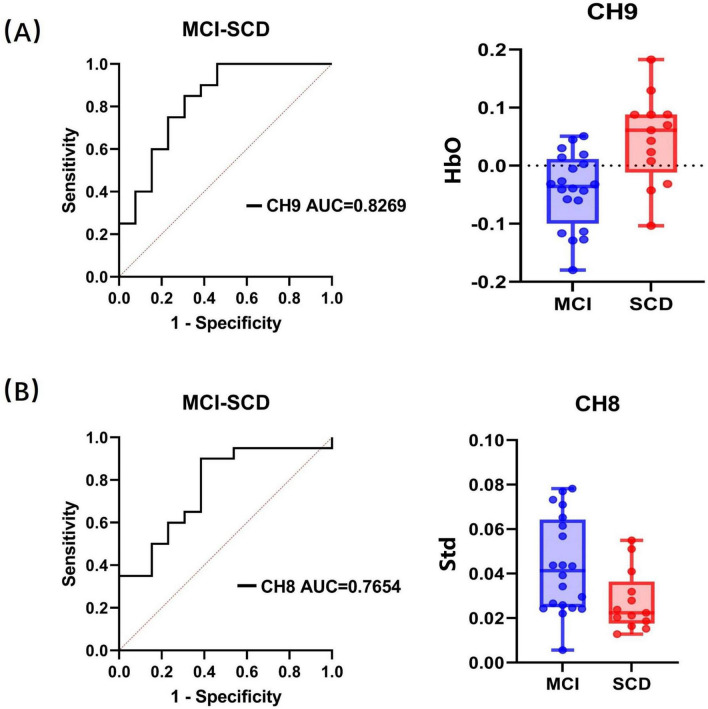
Receiver operating characteristic classification curve and the distribution of significant factors within groups. **(A)** HbO concentration in channel CH9 within 5–15 s. **(B)** Standard deviation in channel CH8 within 20–60 s. “Std” represents the standard deviation of HbO.

The goodness of fit was confirmed through the Hosmer and Lemeshow test for the average HbO concentration of channel CH9 in the 5–15 s window (χ^2^(8) = 9.453, *p* = 0.306) and the HbO concentration standard deviation of channel CH8 in the 20–60 s window (χ^2^(8) = 11.391, *p* = 0.180). The *p* values for both significant predictors were greater than 0.05, indicating a good fit of the model for this classification task. Subsequent ROC analysis revealed area under the curve (AUC) values of 0.8269 (*p* = 0.002) and 0.7654 (*p* = 0.011) for the two predictors, respectively. The ROC curves and the distribution of these significant factors across different groups are illustrated in Figure 5.

Furthermore, the ROC analysis identified cutoff values for the average HbO concentration of channel CH9 in the 5–15 s window (0.519, sensitivity = 0.769, specificity = 0.75) and the HbO concentration standard deviation of channel CH8 in the 20–60 s window (0.515, sensitivity = 0.9, specificity = 0.615). Specifically, since the *t*-value of the two-sample *t*-test for the average HbO concentration of channel CH9 within the 5–15 s window was positive, and for the HbO concentration standard deviation of channel CH8 within the 20–60 s window, it was negative (with SCD as the first group and MCI as the second group), we infer that participants would be classified as SCD when the average HbO concentration of channel CH9 exceeds the cutoff value in the 5–15 s window, and as SCD when the HbO concentration standard deviation of channel CH8 falls below the cutoff value in the 20–60 s window.

Notably, even after accounting for demographic factors such as age, gender, and education level in the statistical model, only the average HbO concentration of channel CH9 in the 5–15 s window and the HbO concentration standard deviation of channel CH8 in the 20–60 s window were selected as significant predictors. This result suggests that these features have an independent association with the differentiation between SCD and MCI beyond demographic factors.

### 3.4 Behavioral performance and fNIRS data analysis during the MemTrax task

#### 3.4.1 Behavioral performance analysis of the MemTrax task

In the MemTrax test, the MCI group exhibited overall poorer performance in picture memory cognition (with a highest correct rate of 98%, lowest correct rate of 52%, and average correct rate of 81.30%, SD = 13.2) compared to the SCD group. Interestingly, the SCD group performed slightly better than the MCI group (with a highest correct rate of 96%, lowest correct rate of 64%, and average correct rate of 86.15%, SD = 8.06). Although the MCI group’s average correct rate was lower than that of the SCD group, a two-sample *t*-test revealed no significant intergroup differences in correct rates (*t*(31) = 1.186, *p* = 0.245). However, in terms of reaction time, the MCI group took slightly longer to complete the same picture memory task compared to the SCD group. Specifically, the SCD group had a maximum reaction time of 1.689 s, a minimum reaction time of 0.855 s, an average reaction time of 1.178 s, and a standard deviation of 0.074. In contrast, the MCI group had a maximum reaction time of 1.865 s, a minimum reaction time of 0.676 s, an average reaction time of 1.175 s, and a standard deviation of 0.062. Despite the slightly better performance of the SCD group in reaction time, the two groups did not show significant intergroup differences in reaction time (*t*(31) = 0.033, *p* = 0.974) based on the statistical analysis. Overall, while statistical results may not indicate significant differences, from a clinical perspective, MCI group members still exhibit inferior performance in everyday life compared to the SCD group.

#### 3.4.2 Hemodynamic data analysis during the MemTrax task

In this study, we analyzed HbO concentration, slope, and standard deviation for both the SCD and MCI groups during the MemTrax task. Due to varying completion times among participants, we uniformly selected the shortest duration of 80 s as the endpoint for data processing. The time windows were still defined according to the VFT standards, dividing the task into four intervals: 5–15, 20–60, 60–80, and 5–80 s, corresponding to the rising, plateau, falling, and overall phases of HbO concentration during the entire cognitive task. Analyzing the average HbO concentration, slope, and standard deviation across these four time windows for each channel, we found no significant intergroup differences (*p* = 0.273–0.926) in these three features across the 19 channels between the SCD and MCI groups. Considering the behavioral performance results during the MemTrax task, it appears that there may be differences in actual daily cognitive function between the two groups, but their performance in these specific channels’ HbO responses did not show statistically significant differences.

## 4 Discussion

The primary objective of this study is to assess the efficacy of fNIRS in distinguishing between patients with SCD and MCI. The study compared the activation patterns of the prefrontal cortex in SCD and MCI patients during task performance under different conditions. The results indicated that, relative to MCI patients, SCD patients exhibited higher levels of FC in the prefrontal cortex during rest. This suggests a relative preservation of cognitive function in SCD. During the initial phase of the VFT, certain prefrontal cortex channels in SCD patients showed significantly higher levels of HbO compared to MCI patients. However, in the latter phase of the task, the differences in HbO levels between the two groups were not significant. Conversely, the standard deviation was lower in SCD patients than in MCI patients within specific channels during 5–15 and 20–60 s time windows. Unfortunately, no distinct features with significant intergroup differences were observed during the MemTrax task, which may be attributed to task difficulty and type. Consequently, this study suggests that fNIRS could serve as a valuable tool for identifying individuals with early-stage dementia.

Previous studies have primarily focused on comparing the hemodynamic activation patterns between normal cognition NC and MCI individuals, with less attention paid to the differences between SCD and MCI. In the existing literature, most studies have focused on the comparison of single features (Niu et al., 2013; Yeung et al., 2016; Yoon et al., 2019). This study, however, takes into account multiple cognitive function indicators. Specifically, the average HbO concentration changes of participants at rest are relatively stable, which is consistent with the research of Yoo et al. (2020) and Ho et al. (2022). But in terms of brain FC, the SCD group showed a higher average FC value than the MCI group (Yeung and Chan, 2020). This suggests that with the deepening of the disease and the decline of cognitive ability, FC may have suffered varying degrees of damage. This phenomenon indicates that changes in FC can be detected in the early stages of AD. This is because FC reflects the ability of different brain regions to work together, and this ability may be impaired early in the pathological process. Interestingly, in certain channels, the FC strength in the MCI group was significantly higher than that in the SCD group, which was contrary to our initial expectations. In studies comparing SCD and NC, it was found that SCD patients exhibit a complex pattern of compensatory neural activity in specific brain regions, particularly in the prefrontal cortex, which is closely associated with higher-order cognitive functions. During the SCD stage, to maintain cognitive performance, some brain regions undergo hyperactivation (Chen et al., 2020). This hyperactivation may, on the one hand, increase the overall connectivity within certain areas, but on the other hand, it may lead to adaptive changes in specific connections related to the prefrontal cortex, manifesting as decreased or disrupted connectivity, which becomes more pronounced as the disease progresses (Lee D. et al., 2023). This change reflects a mixed pattern, where some connections weaken due to the redistribution of neural resources or the shifting of task-related functions, while others may show enhanced activation. Some researchers have also observed a similar contradictory trend in their results, as we did (Lee T. L. et al., 2023). We hypothesize that the functional connections in the SCD group with lower connectivity strength compared to the MCI group likely follow a similar compensatory mechanism. As SCD progresses to MCI, patients in the MCI group exhibit more widespread abnormalities in brain FC. At this stage, some regions continue to attempt compensation for emerging deficits through compensatory activity, potentially resulting in abnormally elevated connectivity values that exceed those observed in the SCD group. Meanwhile, other regions, due to disease progression, begin to show early signs of functional impairment, leading to reduced brain FC strength. This requires further experimental validation. Besides, no significant group differences were found in the average HbO concentration and its derived features. This may be because in the early stages of the disease, the HbO signal has not yet mutated or the changes are not obvious and are not easily detected.

During the VFT, there were large fluctuations in the frontal lobe activation patterns of different group participants. This mainly comes from the average HbO concentration and standard deviation in the 5–15 s time window and the average HbO standard deviation in the 20–60 s time window. Specifically, the average HbO concentration of the SCD group on specific frontal lobe channels was significantly higher than that of the MCI group, and this difference was more obvious within 5–15 s of the task start. This indicates that at the beginning of the VFT, the SCD group can better mobilize cognitive resources when the cognitive task starts, while the MCI group may lack sufficient cognitive resources to quickly meet the cognitive demands of the cognitive task (Yoon et al., 2019; Butters et al., 2023). But in the later stages of the task, there was no significant group difference in the HbO level between the SCD and MCI groups. Since the difficulty of the task and the group will also have a certain interaction effect on the HbO level (Yeung et al., 2016). We speculate that this may be due to the insufficient difficulty of the task causing the participants of the SCD and MCI groups to not reach a sufficient level of difference, so there is no significant difference in the HbO level. Task difficulty is an important factor for the sensitivity of brain function measurement and needs to be given more attention and discussion in future research. In addition, the HbO standard deviation of the SCD group within 5–15 and 20–60 s is also lower than that of the MCI group. This indicates that during the execution of the task, the fluctuation of the HbO level of the SCD group is smaller and more stable. The higher standard deviation of the MCI group may reflect larger fluctuations in brain activity, resulting in more unstable neural activity and hemodynamic responses. The main reason for this situation is that a larger hemodynamic response often increases the standard deviation of HbO in the time window (Keles et al., 2021; Keles et al., 2022). Therefore, during the cognitive task, frequent brain activity caused this phenomenon. Interestingly, the average HbO concentration in the 20–60 s time window did not show any significant differences. We speculate that this may be due to the large fluctuations in the HbO level in the later stages of the task. Even if a large hemodynamic response occurs, the average HbO value in the window is still close to a certain constant value. Therefore, in addition to the analysis of HbO concentration and FC, the standard deviation of HbO is also a commonly used indicator in fNIRS signal research, which helps to reveal the stability and variability of brain activity (Tai and Chau, 2009; Holper and Wolf, 2011; Aghajani et al., 2017). This information is crucial for understanding the differences in brain function and hemodynamic responses of SCD and MCI patients during cognitive tasks. Regrettably, in terms of MemTrax, although the SCD group performed better than the MCI group in the task, there was no significant group difference. We also have not yet found any significant group difference features in terms of hemodynamic responses. We speculate that this may be due to the low difficulty setting of the task, which did not reach the cognitive load of MCI patients. Therefore, the expected difference cannot be observed at the HbO level. In addition, it may also be related to the hemodynamic activation pattern of SCD and MCI and the cognitive domain of the task undertaken. Therefore, when designing future research, considering the importance of task difficulty and type for identifying the cognition and hemodynamic responses of SCD and MCI patients, it may be necessary to design more diversified and challenging tasks. This needs further research to explore. In summary, these findings suggest that individuals with MCI may exhibit abnormal brain activation before objective cognitive impairments become apparent. This indicates that SCD may represent a pre-stage of MCI, providing important clues for the early detection and intervention strategies of AD.

## 5 Conclusion

This study utilized fNIRS technology to reveal differences in prefrontal cortex activation patterns between patients with SCD and MCI within specific time windows. Comparative analysis of hemodynamic activation patterns in the prefrontal cortex during resting state and specific cognitive tasks (such as the Verbal Fluency Test, VFT) revealed that SCD patients had higher average levels of prefrontal FC during rest than MCI patients, suggesting a relative preservation of cognitive functions in SCD. Additionally, during the initial phase of the VFT, SCD patients exhibited significantly higher average concentrations of HbO in certain channels, indicating better mobilization of cognitive resources at the onset of the task. However, as the task progressed, the differences in HbO levels between the groups became insignificant, which may be related to task difficulty and individual differences in cognitive resources. Notably, the SCD group had lower HbO standard deviation within specific time windows, indicating smaller fluctuations in brain activity and more stable neural and hemodynamic responses during task execution. Nevertheless, no significant hemodynamic differences were observed between the groups during the MemTrax task, which may be associated with task design, difficulty settings, or relevance to specific cognitive domains. Future research needs to design more challenging tasks to more accurately identify SCD and MCI patients. This indicates that fNIRS may be considered a potential biomarker for the early detection of SCD, which is crucial for early identification and intervention in AD.

## Data Availability

The raw data supporting the conclusions of this article will be made available by the authors, without undue reservation.

## Footnotes

1
https://aaic.alz.org/nia-aa.asp
